# Associations of chronic lung disease, insufficient sleep, and pain with incident frailty in Chinese middle-aged and older adults

**DOI:** 10.4178/epih.e2026016

**Published:** 2026-04-21

**Authors:** Wenyan Hu, Lifang Chen, Tingyan Li, Yongping Gu

**Affiliations:** 1 General Practice, Linping Campus, Second Affiliated Hospital, Zhejiang University School of Medicine, Hangzhou, China; 2 Pulmonary and Critical Care Medicine, Linping Campus, Second Affiliated Hospital, Zhejiang University School of Medicine, Hangzhou, China

**Keywords:** Lung disease, Sleep deprivation, Pain, Frailty

## Abstract

**OBJECTIVES:**

Chronic lung disease, insufficient sleep, and chronic pain are prevalent among middle-aged and older adults and may synergistically accelerate frailty. This study was conducted to examine the independent and combined associations of chronic lung disease, insufficient sleep, and pain with frailty risk among middle-aged and older Chinese adults.

**METHODS:**

This study used data from the China Health and Retirement Longitudinal Study. Multivariable logistic regression and mediation analyses were used to examine the associations among chronic lung disease, insufficient sleep, pain, and frailty. Pain was categorized into any bodily pain and chest pain, with the latter analyzed separately due to its potential relationship with chronic lung disease. Subgroup analyses stratified by age and sex were also conducted to assess effect modification.

**RESULTS:**

Chronic lung disease combined with insufficient sleep (odds ratio [OR], 2.71; 95% confidence interval [CI], 1.70 to 4.22), pain (OR, 3.04; 95% CI, 1.86 to 4.86), or chest pain (OR, 2.58; 95% CI, 1.07 to 5.56) was significantly associated with higher odds of frailty. Subgroup analyses showed an interaction among chronic lung disease, chest pain, and age (p for interaction <0.05). Mediation analysis indicated that sleep, pain, and chest pain mediated 10.1%, 26.2%, and 14.0% of the association between chronic lung disease and frailty.

**CONCLUSIONS:**

The combined presence of chronic lung disease with insufficient sleep or pain was associated with an increased risk of incident frailty. Screening and interventions targeting these factors may help mitigate frailty among middle-aged and older adults.

## GRAPHICAL ABSTRACT

[Fig f4-epih-48-e2026016]

## Key Message

There remains an unmet need for integrated screening and management of insufficient sleep and pain especially chest pain in middle-aged and older adults with chronic lung disease to mitigate frailty risk. This study reveals that the coexistence of chronic lung disease with either short sleep or pain significantly amplifies new-onset frailty risk with systemic pain alone mediating 26.2 percent of the association. These findings underscore the value of multimodal early interventions targeting modifiable comorbidities to prevent or delay frailty in aging populations with respiratory disease.

## INTRODUCTION

Frailty is a multisystem clinical syndrome characterized by increased vulnerability and reduced resistance to stressors due to diminished physiological reserve and functional decline. This condition represents a non-specific age-related state [1]. As a critical transitional stage between health and disability, frailty not only impairs the ability to perform daily activities and reduces quality of life but also substantially increases the risk of adverse outcomes, including falls, cognitive impairment, hospitalization, and mortality [2,3]. With the rapid aging of China’s population, frailty has emerged as a major public health challenge. Epidemiological data indicate that the prevalence of frailty among middle-aged and older Chinese adults is 16.3%, and the risk increases markedly with age, imposing substantial burdens on healthcare resources, families, and society [4,5]. In this context, identifying risk factors for frailty is essential for precision prevention and targeted interventions.

Chronic lung disease (CLD), including chronic obstructive pulmonary disease (COPD), chronic bronchitis, asthma, and interstitial lung disease, is a major public health concern among middle-aged and older adults [6]. Extensive epidemiological studies have shown a robust association between CLD and frailty, likely because they share risk factors, such as advanced age and smoking, as well as pathophysiological mechanisms, including chronic inflammation, immune dysfunction, and neuroendocrine dysregulation [7-9]. Current evidence indicates that COPD, chronic bronchitis, and idiopathic pulmonary fibrosis are associated with an elevated risk of frailty [10-12], and patients with COPD have approximately twice the frailty risk of those without COPD [13]. Symptom burden and functional limitations related to CLD may also vary by sex. For example, female patients with CLD appear to have a higher disease burden and may be more susceptible to reduced quality of life and frailty [14].

Furthermore, clinical profiles of patients with CLD indicate a high prevalence of comorbid symptoms, particularly sleep problems and chronic pain. Meta-analyses have reported that 21.6–29.5% of patients with COPD experience sleep problems [15], exceeding rates in the general population, whereas chronic pain affects up to 85% of patients with CLD, far surpassing general-population rates [16]. Notably, both symptoms are independent risk factors for frailty. A cross-sectional study reported that insufficient sleep (IS) was positively associated with frailty and with 2.62-fold higher odds of mild frailty among older adults [17]. A systematic review indicated that both acute and chronic sleep deprivation can decrease muscle strength, explosive power, and muscle endurance, leading to impaired neuromuscular function and increased fatigue [18]. Similarly, a prospective community-based cohort study of older adults found that moderate to severe chronic pain was associated with 1.13-fold higher odds of frailty [19]. Another study also reported a positive association between pain and frailty, with generalized pain associated with approximately 3.3-fold and 4.7-fold higher odds of pre-frailty and frailty, respectively [20].

Among CLD-related pain symptoms, chest pain can be more closely linked to respiratory function and may impose more immediate limitations on mobility and physiological reserve. Previous studies have suggested that chest pain not only reflects the burden of underlying cardiopulmonary disease but also is closely associated with activity limitation, reduced exercise tolerance, and impaired quality of life [21,22]. These factors represent important mediating links in the development and progression of frailty. Collectively, these findings suggest potential mechanisms underlying frailty risk in patients with CLD and highlight possible targets for clinical intervention. However, the independent and combined effects of these symptoms on the risk of frailty in the CLD population remain unclear. In addition, the role of chest pain, as a key component of CLD-related pain, in the development of frailty has not been fully explored.

To address this gap, this study used data from the China Health and Retirement Longitudinal Study (CHARLS) to systematically investigate the associations of CLD and its comorbid symptoms with frailty risk among middle-aged and older adults in China. In addition, given the potential differences in the relationships of CLD and its comorbidities with frailty across age and sex groups, this study aimed to examine heterogeneity across age and sex strata through subgroup analyses. These analyses may provide empirical evidence to help identify high-risk subgroups and inform targeted strategies for frailty prevention and intervention.

## MATERIALS AND METHODS

### Study design and population

CHARLS is an ongoing, nationally representative longitudinal survey of adults aged 45 years and older (https://charls.pku.edu.cn/en/). This survey uses a multistage stratified probability-proportional-to-size sampling design, with rural villages and urban communities serving as the primary sampling units. The survey collects data on socio-demographic characteristics, economic status, health status, and functioning. Blood sample data were collected in 2011 and 2015 [23].

**Figure 1.**
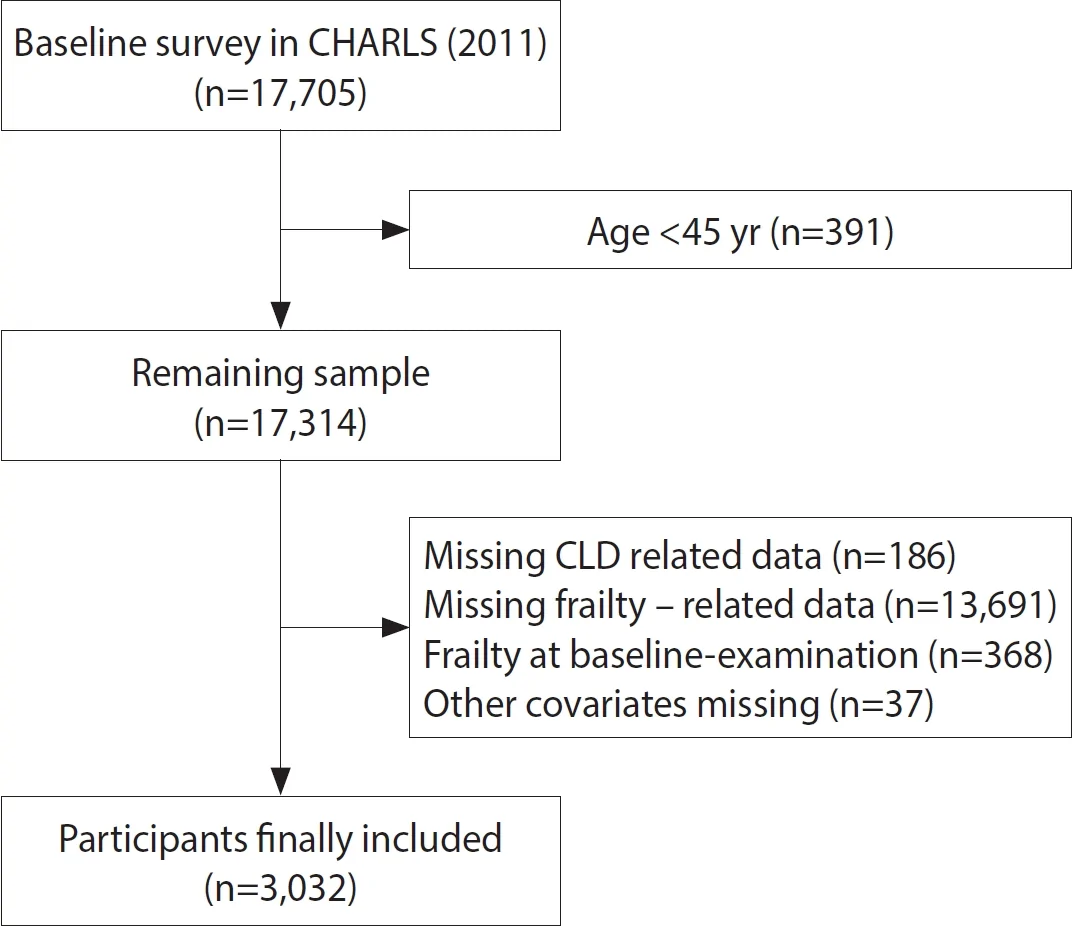
Inclusion and exclusion criteria. CHARLS, China Health and Retirement Longitudinal Study; CLD, chronic lung disease.

In this study, frailty was defined based on physical examinations and objective functional measurements collected systematically during the 2011–2012, 2013–2014, and 2015–2016 CHARLS cycles. Therefore, only data from the corresponding survey waves in 2011, 2013, and 2015 were included in the analysis. Participants were excluded if they were younger than 45 years (n=391), had missing data on CLD-related variables (n=186), lacked frailty-related variables (n=13,691), had pre-existing frailty at baseline (n=368), or had missing data on other covariates (n=37). After these exclusions, 3,032 participants were included. The selection process is illustrated in Figure 1.

### Data collection and definitions

CLD was defined based on self-reported physician diagnosis using the following CHARLS question: “Have you been diagnosed with [chronic lung diseases, such as chronic bronchitis, emphysema (excluding tumors, or cancer)] by a doctor?”

Sleep duration was obtained from the lifestyle and health behavior section of the CHARLS questionnaire. Specifically, participants were asked, “During the past month, what was your actual sleep duration at night (average hours per night)?”

Pain was assessed using the following questions: “Are you often troubled by any bodily pain (‘No’ or ‘Yes’)?” and “Which part of your body feels pain? Please list all the body parts where you currently feel pain.” Respondents who reported no pain in any body part were classified as having no pain. Chest discomfort, including chest pain, is relatively common among patients with lung disease and may relate more directly to pulmonary pathology and impaired cardiopulmonary function than pain in broader regions such as the limbs or lower back. Accordingly, chest pain was extracted separately for analysis. Based on the second question referenced above, respondents who reported pain in the chest area (e.g., chest or chest cavity) were classified as having chest pain.

We further combined CLD with sleep status, pain, and chest pain and categorized participants as follows: (1) no CLD and enough sleep (ES); (2) CLD and ES; (3) no CLD and IS; (4) CLD and IS; (5) no CLD and no pain; (6) CLD and no pain; (7) no CLD and pain; (8) CLD and pain; (9) no CLD and no chest pain; (10) CLD and no chest pain; (11) no CLD and chest pain; and (12) CLD and chest pain.

Nighttime sleep duration was self-reported by participants in response to the CHARLS question regarding their average hours slept per night in the past month. Sleep duration was then categorized as IS (≤6 hours) or ES (>6 hours) according to the reported response [24,25].

In this study, frailty was defined using the classic frailty phenotype proposed by Fried et al. [26] in combination with the revised CHARLS index. In the original study by Fried et al. [26], the frailty phenotype was based on involuntary weight loss, self-reported exhaustion, reduced grip strength, slowed walking speed, and low physical activity. This phenotype independently predicted adverse outcomes, including falls, functional limitations, hospitalization, and death during follow-up, demonstrating good concurrent and predictive validity. Subsequently, a revised standard [27] was developed for the CHARLS database based on the framework of Fried et al. [26] and has shown acceptable validity and reliability for identifying frailty in middle-aged and older adults in China. Therefore, this approach was considered appropriate for assessing frailty in the present study. Frailty was treated as a binary outcome and was assessed as follows: (1) Weakness was assessed using the self-reported item “difficulty lifting or carrying more than 5 kg.” (2) Slowness was considered present if participants reported difficulty walking 100 m or climbing several flights of stairs without resting. (3) Exhaustion was considered present if participants responded “3–4 days” or “5–7 days” per week to either of the following items from the Chinese version of the Center for Epidemiologic Studies Depression (CES-D) scale: “I felt everything I did was an effort” or “I could not get going.” (4) Low physical activity was defined as not engaging in physical activity or walking for at least 10 minutes at a time during a usual week. (5) Weight loss was defined as an unintentional loss of 5 kg or more in the past year or a current body mass index ≤18.5 kg/m2, calculated as weight in kilograms divided by the square of height in meters.

Frailty was defined as the presence of 3 or more of these 5 components.

In this study, incident frailty was defined operationally as follows: participants did not meet the frailty criteria at baseline in 2011 but were classified as frail in either follow-up survey (2013 or 2015).

### Covariates

Covariates included age, sex, comorbidities, and other clinically relevant characteristics. Additional details are provided in the Results section.

### Statistical analysis

Baseline characteristics were summarized according to frailty status. Categorical variables are presented as numbers and percentages (n [%]), and continuous variables are expressed as mean±standard deviation. Group differences were assessed using analysis of variance for continuous variables and the chi-square test for categorical variables. Multivariable logistic regression was first used to evaluate the association between CLD and frailty and the joint associations of CLD and sleep, pain, and chest pain with frailty. Interaction analyses were then performed to assess interactions between CLD and sleep, pain, and chest pain, and subgroup analyses examined whether the associations of CLD, sleep, pain, and chest pain with frailty differed by age and sex. Subgroup analyses were conducted using the jstable package, and interaction analyses were performed with the epiR package. The 3 interaction measures—relative excess risk due to interaction (RERI), attributable proportion (AP), and synergy index (SI)—were derived from the regression coefficients and covariance matrices from the multivariable logistic regression models. A 95% confidence interval (CI) for RERI or AP that included 0, or for SI that included 1, was considered to indicate no additive interaction. The mediating effects of sleep, pain, and chest pain were examined using the bootstrap method with 500 resampling iterations. A p-value <0.05 was considered to indicate statistical significance. Mediation analyses were performed using the mediation package. Model 1 was unadjusted. Model 2 was adjusted for age, sex, education level, and marital status. Model 3 was further adjusted for smoking, alcohol consumption, hypertension, dyslipidemia, and diabetes. All analyses were performed in R version 4.1.3 (R Foundation for Statistical Computing, Vienna, Austria).

### Ethics statement

Ethics approval and informed consent were not required because this study used publicly available data.

## RESULTS

### Baseline characteristics

A total of 3,032 participants were included. Table 1 presents the baseline demographic and clinical characteristics. The mean age of the participants was 58.5±8.8 years, and 52.9% were female. Compared with the non-frail group, the frail group tended to be older, included a higher proportion of female participants, had lower educational attainment, had a higher proportion of participants who were single, and had higher prevalence rates of both hypertension and CLD. However, the frail group had a lower prevalence of alcohol use than the non-frail group (all p<0.05).

### Independent and combined associations of chronic lung disease, insufficient sleep, pain, and chest pain with frailty risk

Table 2 presents the associations of CLD with frailty, as well as the combined associations of CLD with sleep status, pain, and chest pain in relation to frailty risk. Across all 3 models, CLD was significantly associated with higher odds of frailty (p<0.05). In the analysis of the combined effects of CLD and sleep status, participants without CLD who had IS and those with CLD and IS had 1.47-fold (odds ratio [OR], 1.47; 95% CI, 1.10 to 1.97; p=0.010) and 2.71-fold (OR, 2.71; 95% CI, 1.70 to 4.22; p<0.001) higher odds of frailty, respectively, than those with no CLD and ES. In the joint analysis of CLD and pain status, participants with no CLD and pain and those with CLD and pain had 2.11-fold (OR, 2.11; 95% CI, 1.58 to 2.82; p<0.001) and 3.04-fold (OR, 3.04; 95% CI, 1.86 to 4.86; p<0.001) higher odds of frailty, respectively, than those with no CLD and no pain. In the joint analysis of CLD and chest pain status, participants with CLD and no chest pain, no CLD and chest pain, and CLD and chest pain had 1.66-fold (OR, 1.66; 95% CI, 1.09 to 2.46; p=0.015), 1.94-fold (OR, 1.94; 95% CI, 1.14 to 3.14; p=0.010), and 2.58-fold (OR, 2.58; 95% CI, 1.07 to 5.56; p=0.022) higher odds of frailty, respectively, than those with no CLD and no chest pain.

Additional interaction analyses presented in Supplementary Materials 1-3 suggested statistically significant additive or multiplicative interactions between CLD and pain in relation to frailty risk, indicating potential synergistic effects between these factors.

### Subgroup analysis results

Stratified analyses (Supplementary Materials 4-6) examined the associations of CLD combined with IS, pain, or chest pain with frailty risk across age and sex subgroups. A statistically significant interaction by age was observed for the association between CLD combined with chest pain and frailty (p for interaction <0.05) (Supplementary Material 6). Most of the examined combinations were associated with significantly higher frailty risk across the subgroups.

### Mediation analysis findings

**Figure 2.**
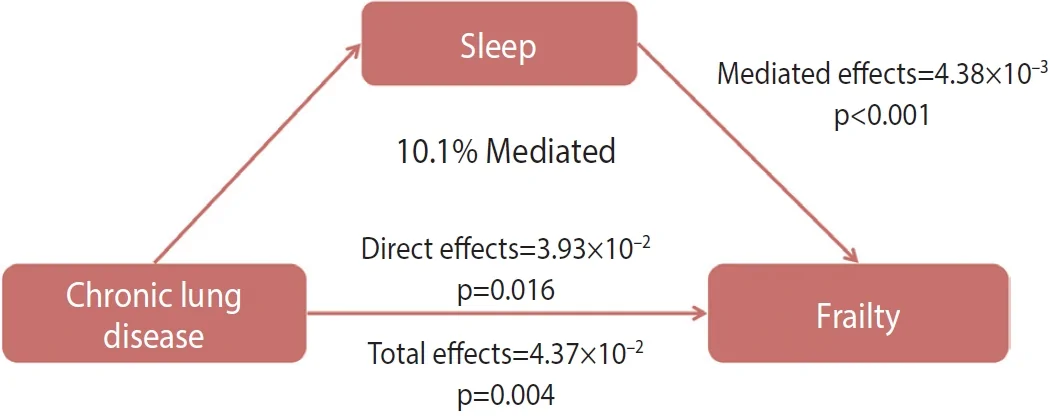
Mediating role of sleep in the association between chronic lung disease and frailty.

**Figure 3.**
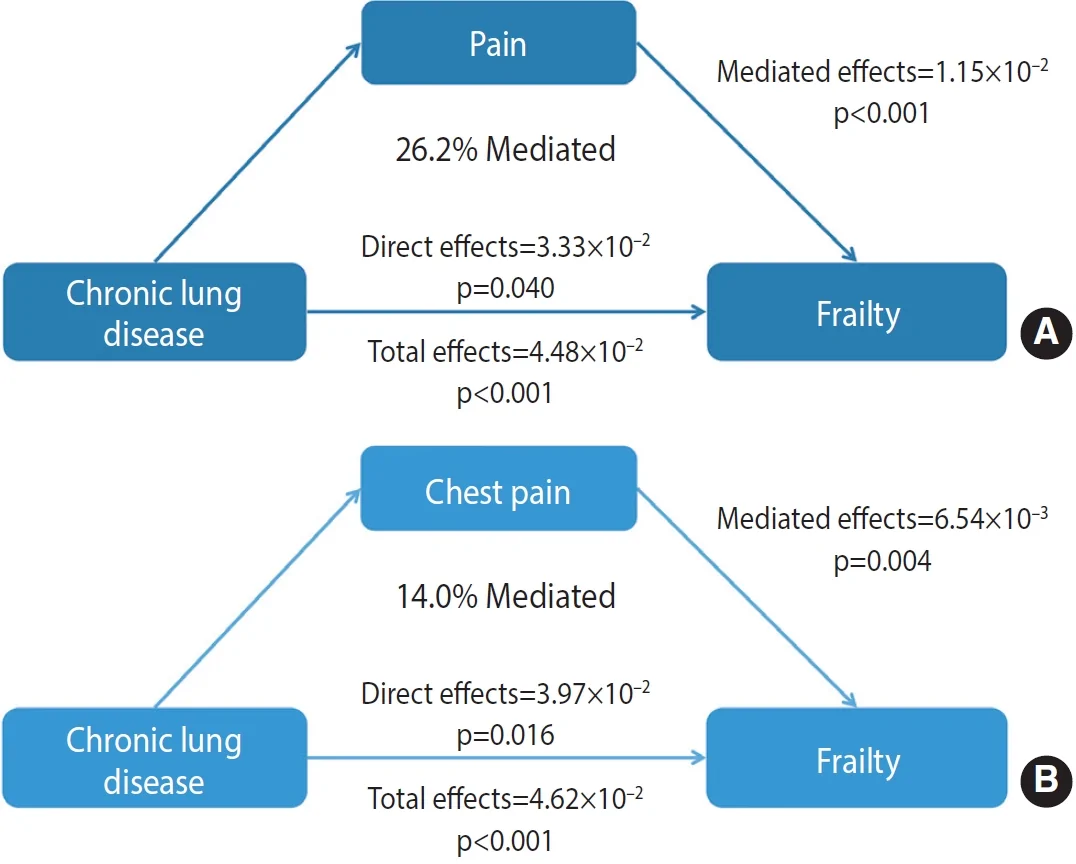
Mediating roles of (A) pain and (B) chest pain in the association between chronic lung disease and frailty.

To further examine the potential mechanisms through which CLD, IS, pain, and chest pain were associated with frailty risk, path analyses were conducted. Mediation analyses using bias-corrected bootstrap methods were then performed to examine these relationships (Figures 2 and 3). As shown in Figure 2, approximately 10.1% of the association between CLD and frailty could be attributed to IS. Figure 3A indicates that approximately 26.2% of the association between CLD and frailty was mediated by pain. Figure 3B shows that approximately 14.0% of this association was mediated by chest pain.

## DISCUSSION

This study systematically evaluated the associations of CLD, IS, pain, and chest pain with frailty risk using CHARLS data while also examining the combined effects of CLD with these coexisting conditions and their differential associations across subgroups. The findings indicate that the co-occurrence of CLD with IS, pain, or chest pain was associated with higher odds of frailty among middle-aged and older adults, with sleep, pain, and chest pain acting as mediators of the association between CLD and frailty.

Consistent with previous research, this study found robust associations of CLD, sleep, and pain with frailty, and these findings remained stable after adjustment for multiple covariates. This is consistent with systematic reviews documenting well-established associations of CLD, sleep, and pain with frailty [28-30]. Epidemiological evidence indicates that frailty is markedly more prevalent among middle-aged and older adults with COPD than among controls (46.2 vs. 22.4%) [31], and similar patterns have been reported for other forms of CLD, including chronic bronchitis and interstitial lung disease, with an 82% prevalence of pre-frailty/frailty [32]. Dyspnea has been identified as a key determinant of frailty among patients with CLD, with each 1-point increase in dyspnea severity associated with approximately doubled odds of pre-frailty/frailty after comprehensive adjustment [33]. These findings suggest that respiratory symptom management may represent an important target for frailty prevention.

Abnormal sleep duration, especially IS, has been closely associated with frailty risk. Multiple studies in older populations have shown that short sleep duration is significantly associated with a higher prevalence of frailty, and some cohort studies have reported that the risk of frailty among short sleepers is approximately 1.44 times to 2.0 times higher [34,35]. Among patients with CLD, shortened sleep duration is more common than among those without CLD and is often accompanied by more frequent apnea and daytime sleepiness [36]. Such long-term sleep deficiency may accelerate the development of frailty by exacerbating systemic inflammation, promoting declines in muscle mass and strength, and impairing energy metabolism and recovery capacity [18,37]. In the present mediation analysis, sleep explained approximately 10.1% of the association between CLD and frailty. In addition, a significant synergistic effect was observed between IS and CLD, with participants who had both CLD and IS exhibiting approximately 2.71-fold higher odds of frailty than those with no CLD and ES. These findings suggest that, in efforts to prevent and manage frailty in patients with CLD, particular attention should be paid to the modifiable risk factor of IS and its combined effect with underlying lung disease.

Chronic pain represents another important mediator. Epidemiological studies have reported a prevalence of 51.8% among patients with COPD, with pain primarily involving the back and joints [38-40]. This widespread pain may contribute to frailty through multiple pathways, including activity limitation, sleep disruption, and exacerbation of inflammation [41]. The mediation analyses in the present study showed that pain accounted for a significant indirect component of the association between CLD and frailty. Chest pain, as a potentially respiratory-related symptom, may be particularly relevant; chest pain has been reported in 54.4% of patients with COPD, with 3 characteristic subtypes: intercostal muscle strain, pleuritic pain, and biomechanical pain [42,43]. Compared with peripheral pain, chest pain may have more direct respiratory implications and could therefore contribute to frailty through more complex pathways.

Clinical evidence also supports interventions targeting these mediators. Pulmonary rehabilitation programs that incorporate respiratory muscle training have shown efficacy in patients with CLD who experience chest pain [44,45]. Pharmacologically, some studies have suggested that, when used appropriately and within recommended doses, acetaminophen or ibuprofen may be associated with a reduced risk of CLD exacerbation [46,47]. For patients with CLD and IS, cognitive behavioral therapy has been associated with durable improvements in sleep, fatigue, and dyspnea lasting at least 3 months [48,49]. Together, these findings may help inform strategies for preventing and managing frailty in patients with CLD.

Unlike conventional cross-sectional studies that describe associations at a single time point, this study used data from multiple CHARLS waves to examine the longitudinal association pattern between CLD, its comorbidities, and incident frailty. In addition, unlike most longitudinal studies that focus on a single exposure or outcome, this study systematically constructed a multivariable analytical framework incorporating CLD, comorbidities including sleep and pain, and frailty, thereby revealing the multifactorial nature of the associations between CLD and frailty. Several limitations should be noted. First, because the physical examination data and objective functional indicators required for frailty assessment were collected systematically only during the 2011–2016 cycles, only data from 2011–2015 were included, which may limit the generalizability of the findings to more recent populations. Second, the assessment of CLD, pain, and sleep duration relied mainly on self-report and lacked objective measurement, which may have introduced recall bias and led to underestimation or overestimation of the observed associations. Third, the mediation analysis used CLD (exposure) and IS/pain (mediators) measured at baseline in 2011 to examine their associations with incident frailty occurring during follow-up in 2013 or 2015. Although this design can help explore potential pathways through which CLD may be associated with frailty through sleep or pain, the concurrent measurement of the exposure and mediators does not allow their temporal sequence to be strictly established. Therefore, these analyses primarily describe patterns of association among the variables, and causal inferences should be made cautiously and verified in future studies with repeated measurements across multiple time points. Finally, the generalizability of these findings beyond Chinese populations remains to be established.

CLD, IS, and pain were each independently associated with frailty and also appeared to interact synergistically, producing combined effects greater than their individual associations. Mediation analyses quantified these relationships and identified IS, pain, and chest pain as significant mediators. These findings underscore the need for integrated clinical strategies that simultaneously address CLD, sleep, and pain to help prevent frailty progression. Future research should clarify the molecular mechanisms underlying these interactions to inform precision interventions.

## Figures and Tables

**Figure f4-epih-48-e2026016:**
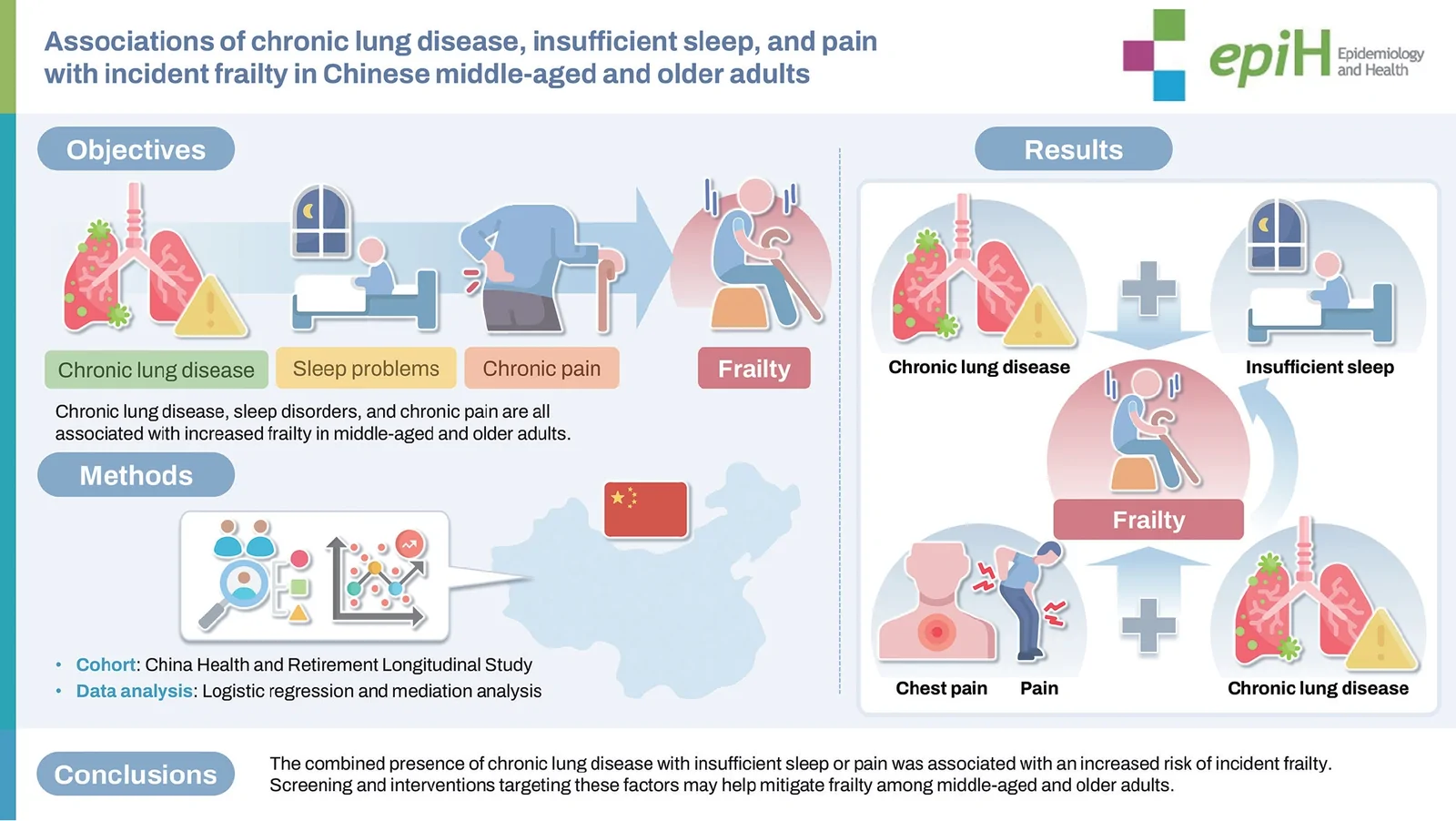


**Table 1.** General characteristics of the study population

Characteristics	Overall	No frailty	Frailty	p-value
Total (n)	3,032	2,779	253	
Age, mean±SD (yr)	58.5±8.8	58.1±8.6	63.1±9.2	<0.001
Sex				<0.001
Female	1,603 (52.9)	1,445 (52.0)	158 (62.5)	
Male	1,429 (47.1)	1,334 (48.0)	95 (37.5)	
Education level				<0.001
Illiterate	791 (26.1)	676 (24.3)	115 (45.5)	
Elementary and below	1,242 (41.0)	1,137 (40.9)	105 (41.5)	
Middle and above	999 (32.9)	966 (34.8)	33 (13.0)	
Marital status				<0.001
Single	334 (11.0)	282 (10.1)	52 (20.6)	
Married	2,698 (89.0)	2,497 (89.9)	201 (79.4)	
Smoking				0.808
No	1,878 (61.9)	1,719 (61.9)	159 (62.8)	
Yes	1,154 (38.1)	1,060 (38.1)	94 (37.2)	
Alcohol consumption				<0.001
No	2,046 (67.5)	1,845 (66.4)	201 (79.4)	
Yes	986 (32.5)	934 (33.6)	52 (20.6)	
Hypertension				<0.001
No	1,857 (61.2)	1,731 (62.3)	126 (49.8)	
Yes	1,175 (38.8)	1,048 (37.7)	127 (50.2)	
Dyslipidemia				0.932
No	1,856 (61.2)	1,700 (61.2)	156 (61.7)	
Yes	1,176 (38.8)	1,079 (38.8)	97 (38.3)	
Diabetes				0.150
No	2,668 (88.0)	2,453 (88.3)	215 (85.0)	
Yes	364 (12.0)	326 (11.7)	38 (15.0)	
CLD				<0.001
No	2,737 (90.3)	2,526 (90.9)	211 (83.4)	
Yes	295 (9.7)	253 (9.1)	42 (16.6)	

Values are presented as number (%). SD, standard deviation; CLD, chronic lung disease.

**Table 2.** Logistic regression analysis of the combined associations of CLD and sleep, pain, and chest pain with frailty^1^

Variables	Model 1	p-value	Model 2	p-value	Model 3	p-value
CLD	1.99 (1.38, 2.81)	<0.001	1.79 (1.23, 2.56)	0.002	1.70 (1.16, 2.44)	0.005
Sleep status						
No CLD and ES	1.00 (reference)		1.00 (reference)		1.00 (reference)	
CLD and ES	1.23 (0.56, 2.39)	0.565	1.17 (0.53, 2.29)	0.680	1.12 (0.51, 2.21)	0.760
No CLD and IS	1.48 (1.11, 1.96)	0.007	1.43 (1.07, 1.91)	0.016	1.47 (1.10, 1.97)	0.010
CLD and IS	3.31 (2.12, 5.07)	<0.001	2.83 (1.79, 4.40)	<0.001	2.71 (1.70, 4.22)	<0.001
Pain status						
No CLD and no pain	1.00 (reference)		1.00 (reference)		1.00 (reference)	
CLD and no pain	1.85 (1.01, 3.17)	0.034	1.69 (0.91, 2.93)	0.079	1.57 (0.84, 2.74)	0.134
No CLD and pain	2.29 (1.73, 3.04)	<0.001	2.10 (1.58, 2.81)	<0.001	2.11 (1.58, 2.82)	<0.001
CLD and pain	3.81 (2.36, 5.97)	<0.001	3.16 (1.93, 5.02)	<0.001	3.04 (1.86, 4.86)	<0.001
Chest pain status						
No CLD and no chest pain	1.00 (reference)		1.00 (reference)		1.00 (reference)	
CLD and no chest pain	1.97 (1.32, 2.88)	<0.001	1.75 (1.15, 2.59)	0.007	1.66 (1.09, 2.46)	0.015
No CLD and chest pain	2.20 (1.32, 3.50)	0.002	2.07 (1.22, 3.35)	0.004	1.94 (1.14, 3.14)	0.010
CLD and chest pain	2.89 (1.23, 6.02)	0.008	2.74 (1.14, 5.90)	0.015	2.58 (1.07, 5.56)	0.022

Values are presented as odds ratio (95% confidence interval). CLD, chronic lung disease; ES, enough sleep; IS, insufficient sleep.

1 Model 1: unadjusted; Model 2: adjusted for age, sex, education level, and marital status; Model 3: adjusted for age, sex, education level, marital status, smoking, alcohol consumption, hypertension, dyslipidemia, and diabetes.

## Data Availability

The data and materials used in this study are available from the corresponding author upon reasonable request.

## Supplementary materials

Supplementary Material 1. Interaction effects between CLD and sleep on frailty risk

Supplementary Material 2. Interaction effects between CLD and sleep on frailty risk

Supplementary Material 3. Interaction effects between CLD and chest pain on frailty risk

Supplementary Material 4. Subgroup analysis of combined associations between CLD, sleep, and frailty

Supplementary Material 5. Subgroup analysis of combined associations between CLD, pain, and frailty

Supplementary Material 6. Subgroup analysis of combined associations between CLD, chest pain, and frailty
