# Supplementary material for: Associations of chronic lung disease, insufficient sleep, and pain with incident frailty in Chinese middle-aged and older adults
Source: Epidemiol Health. 2026 Apr 21;48:e2026016. doi: 10.4178/epih.e2026016 (PMC13503062; doi:10.4178/epih.e2026016)
Supplement: Supplementary file 1 [file epih-48-e2026016-Supplementary-1.docx]

Supplementary Materials 1. Interaction effects between CLD and sleep on frailty risk

|  | Interactive effects (95% CI) | | |
| --- | --- | --- | --- |
|  | Model 1 | Model 2 | Model 3 |
| Additive effect |  |  |  |
| RERI | -1.09 (-2.19 ~ 0.02) | 0.70 (-0.19 ~ 1.59) | 0.40 (-0.29 ~ 1.10) |
| AP | -1.30 (-3.19 ~ 0.59) | 0.29 (0.05 ~ 0.53) | 0.21 (-0.06 ~ 0.48) |
| SI | -0.180 (Na ~ Na) | 2.02 (0.98 ~ 4.20) | 1.77 (0.73 ~ 4.29) |
| Multiplicative effect | 0.55 (0.23 ~ 1.22) | 1.72 (1.29 ~ 2.28) | 1.54 (1.15 ~ 2.06) |

RERI: relative excess risk due to interaction; AP: proportion attributable to interaction; SI: synergy index
