# Supplementary material for: Associations of chronic lung disease, insufficient sleep, and pain with incident frailty in Chinese middle-aged and older adults
Source: Epidemiol Health. 2026 Apr 21;48:e2026016. doi: 10.4178/epih.e2026016 (PMC13503062; doi:10.4178/epih.e2026016)
Supplement: Supplementary file 2 [file epih-48-e2026016-Supplementary-2.docx]

Supplementary Materials 2. Interaction effects between CLD and pain on frailty risk

|  | Interactive effects (95% CI) | | |
| --- | --- | --- | --- |
|  | Model 1 | Model 2 | Model 3 |
| Additive effect |  |  |  |
| RERI | 0.67 (-1.27 ~ 2.60) | 3.66 (-0.05 ~ 7.36) | 2.71 (-0.3 ~ 5.73) |
| AP | 0.17 (-0.28 ~ 0.63) | 0.57 (0.41 ~ 0.73) | 0.50 (0.31 ~ 0.69) |
| SI | 1.31 (0.61 ~ 2.83) | 3.04 (2.13 ~ 4.35) | 2.62 (1.78 ~ 3.86) |
| Multiplicative effect | 0.90 (0.44 ~ 1.91) | 1.82 (1.37 ~ 2.42) | 1.63 (1.22 ~ 2.19) |

RERI: relative excess risk due to interaction; AP: proportion attributable to interaction; SI: synergy index
