# Supplementary material for: Associations of chronic lung disease, insufficient sleep, and pain with incident frailty in Chinese middle-aged and older adults
Source: Epidemiol Health. 2026 Apr 21;48:e2026016. doi: 10.4178/epih.e2026016 (PMC13503062; doi:10.4178/epih.e2026016)
Supplement: Supplementary file 3 [file epih-48-e2026016-Supplementary-3.docx]

Supplementary Materials 3. Interaction effects between CLD and chest pain on frailty risk

|  | Interactive effects (95% CI) | | |
| --- | --- | --- | --- |
|  | Model 1 | Model 2 | Model 3 |
| Additive effect |  |  |  |
| RERI | -0.28 (-2.83 ~ 2.28) | 3.58 (0.16 ~ 7.00) | 2.51 (-0.11 ~ 5.13) |
| AP | -0.10 (-1.04 ~ 0.85) | 0.56 (0.41 ~ 0.71) | 0.49 (0.32 ~ 0.67) |
| SI | 0.87 (0.24 ~ 3.22) | 2.97 (2.11 ~ 4.18) | 2.58 (1.79 ~ 3.71) |
| Multiplicative effect | 0.67 (0.24 ~ 1.73) | 1.77 (1.33 ~ 2.36) | 1.59 (1.19 ~ 2.13) |

RERI: relative excess risk due to interaction; AP: proportion attributable to interaction; SI: synergy index
