# Supplementary material for: Associations of chronic lung disease, insufficient sleep, and pain with incident frailty in Chinese middle-aged and older adults
Source: Epidemiol Health. 2026 Apr 21;48:e2026016. doi: 10.4178/epih.e2026016 (PMC13503062; doi:10.4178/epih.e2026016)
Supplement: Supplementary file 4 [file epih-48-e2026016-Supplementary-4.docx]

Supplementary Materials 4. Subgroup analysis of combined associations between CLD, sleep, and frailty

| Variable | Subgroup | Level | OR (95%CI) | P _(value)_ | P _(interaction)_ |
| --- | --- | --- | --- | --- | --- |
| Age | < 60 years | No CLD and ES | Ref |  | 0.718 |
|  |  | CLD and ES | 1.29(0.37~4.46) | 0.689 |  |
|  |  | No CLD and LS | 1.72(1.10~2.69) | 0.018 |  |
|  |  | CLD and LS | 3.78(1.70~8.40) | 0.001 |  |
|  | ≥ 60 years | No CLD and ES | Ref |  |  |
|  |  | CLD and ES | 1.02(0.41~2.53) | 0.966 |  |
|  |  | No CLD and LS | 1.29(0.88~1.89) | 0.195 |  |
|  |  | CLD and LS | 2.38(1.38~4.12) | 0.002 |  |
| Sex | Female | No CLD and ES | Ref |  | 0.887 |
|  |  | CLD and ES | 1.21(0.45~3.28) | 0.710 |  |
|  |  | No CLD and LS | 1.37(0.95~1.98) | 0.091 |  |
|  |  | CLD and LS | 2.69(1.46~4.96) | 0.002 |  |
|  | Male | No CLD and ES | Ref |  |  |
|  |  | CLD and ES | 1.05(0.35~3.15) | 0.926 |  |
|  |  | No CLD and LS | 1.65(1.02~2.67) | 0.041 |  |
|  |  | CLD and LS | 2.80(1.40~5.57) | 0.003 |  |
