# Supplementary material for: Associations of chronic lung disease, insufficient sleep, and pain with incident frailty in Chinese middle-aged and older adults
Source: Epidemiol Health. 2026 Apr 21;48:e2026016. doi: 10.4178/epih.e2026016 (PMC13503062; doi:10.4178/epih.e2026016)
Supplement: Supplementary file 5 [file epih-48-e2026016-Supplementary-5.docx]

Supplementary Materials 5. Subgroup analysis of combined associations between CLD, pain, and frailty

| Variable | Subgroup | Level | OR (95%CI) | P _(value)_ | P _(interaction)_ |
| --- | --- | --- | --- | --- | --- |
| **Age** | < 60 years | No CLD and No Pain | Ref |  | 0.796 |
|  |  | CLD and No Pain | 2.03(0.68~6.01) | 0.202 |  |
|  |  | No CLD and Pain | 2.51(1.59~3.94) | <0.001 |  |
|  |  | CLD and Pain | 3.82(1.67~8.74) | 0.002 |  |
|  | ≥ 60 years | No CLD and No Pain | Ref |  |  |
|  |  | CLD and No Pain | 1.42(0.70~2.85) | 0.328 |  |
|  |  | No CLD and Pain | 1.87(1.27~2.75) | 0.001 |  |
|  |  | CLD and Pain | 2.84(1.58~5.12) | 0.001 |  |
| **Sex** | Female | No CLD and No Pain | Ref |  | 0.397 |
|  |  | CLD and No Pain | 1.84(0.81~4.18) | 0.145 |  |
|  |  | No CLD and Pain | 1.77(1.23~2.56) | 0.002 |  |
|  |  | CLD and Pain | 2.64(1.39~5.02) | 0.003 |  |
|  | Male | No CLD and No Pain | Ref |  |  |
|  |  | CLD and No Pain | 1.45(0.61~3.44) | 0.397 |  |
|  |  | No CLD and Pain | 2.93(1.80~4.75) | <0.001 |  |
|  |  | CLD and Pain | 3.75(1.80~7.81) | <0.001 |  |
