# Supplementary material for: Associations of chronic lung disease, insufficient sleep, and pain with incident frailty in Chinese middle-aged and older adults
Source: Epidemiol Health. 2026 Apr 21;48:e2026016. doi: 10.4178/epih.e2026016 (PMC13503062; doi:10.4178/epih.e2026016)
Supplement: Supplementary file 6 [file epih-48-e2026016-Supplementary-6.docx]

Supplementary Materials 6. Subgroup analysis of combined associations between CLD, chest pain, and frailty

| Variable | Subgroup | Level | OR (95%CI) | P _(value)_ | P _(interaction)_ |
| --- | --- | --- | --- | --- | --- |
| **Age** | < 60 years | No CLD and No Chest pain | Ref |  | 0.034 |
|  |  | CLD and No Chest pain | 2.19(1.04~4.61) | 0.039 |  |
|  |  | No CLD and Chest pain | 4.19(2.18~8.03) | <0.001 |  |
|  |  | CLD and Chest pain | 2.57(0.73~9.07) | 0.144 |  |
|  | ≥ 60 years | No CLD and No Chest pain | Ref |  |  |
|  |  | CLD and No Chest pain | 1.50(0.92~2.44) | 0.103 |  |
|  |  | No CLD and Chest pain | 0.88(0.38~2.00) | 0.754 |  |
|  |  | CLD and Chest pain | 2.88(0.97~8.53) | 0.056 |  |
| **Sex** | Female | No CLD and No Chest pain | Ref |  | 0.606 |
|  |  | CLD and No Chest pain | 1.75(1.00~3.07) | 0.05 |  |
|  |  | No CLD and Chest pain | 1.54(0.82~2.91) | 0.182 |  |
|  |  | CLD and Chest pain | 2.49(0.86~7.19) | 0.091 |  |
|  | Male | No CLD and No Chest pain | Ref |  |  |
|  |  | CLD and No Chest pain | 1.58(0.86~2.89) | 0.137 |  |
|  |  | No CLD and Chest pain | 3.08(1.35~7.03) | 0.008 |  |
|  |  | CLD and Chest pain | 2.73(0.75~9.91) | 0.128 |  |
